# Supplementary material for: Studies Needed to Address Public Health Challenges of the 2009 H1N1 Influenza Pandemic: Insights from Modeling
Source: PLoS Med. 2010 Jun 1;7(6):e1000275. doi: 10.1371/journal.pmed.1000275 (PMC2879409; doi:10.1371/journal.pmed.1000275)
Supplement: Alternative Language Abstract S1 — Abstract translated into German by DJS. (0.03 MB DOC) [file pmed.1000275.s001.doc]

Inhaltspunkte

- Während sich die Epidemiologie des pandemischen 2009 Grippevirus in 2010 weiter entwickelt, werden sich in den nächsten 12 bis 18 Monaten wesentliche politische Herausforderungen stellen.
- Wir sehen sechs Herausforderungen im Gesundheitswesen voraus und bestimmen, welche Daten  bei der öffentlichen Entscheidungsfindung notwendig sind: das Messen von altersspezifischer Immunität, genaue Quantifikation des Infektionsschweregrads, eine Verbesserung der Ergebnisse in der Behandlung schwerer Fälle, eine Quantifikation der Effektivität der Behandlungen,  ein schnelles Erfassen der  vollständigen Einwirkung der Pandemie auf Mortalität, und die schnelle Erkennug und Reaktion auf antigenische Varianten.
- Repräsentative serologische Umfragen heben sich als kritische Datenquelle heraus, um die Ungewissheit zu reduzieren, die die Entscheidungsfidung bei pharmazeutischen und nicht pharmazeutischen Eingriffen umgibt, nachem sich die erste Woge gelegt hat.
- Die kontinuirliche Nachverfolgung des Inzidenzzeitlaufs schwerer H1N1pdm Fälle wird ein klares Bild der Variabilität der zugrundeliegenden Übertragbarkeit des Virus bei Bevölkerung umfassenden Verhaltensänderungen wie Schulimpfungen oder nicht pharmazeutischen Eingriffen ergeben.
