# Supplementary material for: Studies Needed to Address Public Health Challenges of the 2009 H1N1 Influenza Pandemic: Insights from Modeling
Source: PLoS Med. 2010 Jun 1;7(6):e1000275. doi: 10.1371/journal.pmed.1000275 (PMC2879409; doi:10.1371/journal.pmed.1000275)
Supplement: Alternative Language Abstract S2 — Abstract translated into Portuguese by TdS. (0.03 MB DOC) [file pmed.1000275.s002.doc]

**Resumo**

- A medida que  a epidemiologia mundial da cepa de influenza pandêmica de 2009 evolua em 2010, desafios políticos significativos continuarão a apresentar-se nos próximos 12 a 18 meses.
- Aqui antecipamos seis desafios de saúde pública e identificamos os dados que são necessários para tomar decisões em saúde pública:   medição da imunidade de diferentes grupos etários diante da infecção;  quantificação da gravidade da doença;  melhoramento dos resultados do tratamento para os casos graves;  quantificação da eficácia das intervenções aplicadas;  medição  do impacto total da pandemia na mortalidade;  e identificação e resposta rápida a variações antigênicas.
- Pesquisas sorológicas representativas destacam-se como uma fonte importante de dados para reduzir a incerteza sobre políticas para as intervenções farmacêuticas e não farmacêuticas após  a onda pandêmica inicial haja passado.
- A vigilância contínua da evolução temporal da incidência de casos graves H1N1pdm dará uma imagem clara da variabilidade subjacente na transmissibilidade do vírus durante mudanças de comportamento na população, como as férias  escolares e outras intervenções não farmacêuticas.
