# Supplementary material for: Studies Needed to Address Public Health Challenges of the 2009 H1N1 Influenza Pandemic: Insights from Modeling
Source: PLoS Med. 2010 Jun 1;7(6):e1000275. doi: 10.1371/journal.pmed.1000275 (PMC2879409; doi:10.1371/journal.pmed.1000275)
Supplement: Alternative Language Abstract S3 — Abstract translated into Spanish by TdS. (0.03 MB DOC) [file pmed.1000275.s003.doc]

**Resumen**

- Conforme la epidemiología mundial de la cepa de influenza pandémica de 2009 evolucione en el 2010, se continuarán presentando retos significativos de política en los próximos 12 a 18 meses.
- Aquí hemos anticipado seis desafíos de salud pública e identificado los datos que se requieren para tomar decisiones en salud pública: medición de la inmunidad de diferentes grupos etáreos frente a la infección; cuantificación de la gravedad de la enfermedad; mejoramiento de los resultados del tratamiento para los casos graves; cuantificación de la eficacia de las intervenciones aplicadas; captura del impacto total de la pandemia en la mortalidad; e identificación y respuesta rápida a variaciones antigénicas.
- Encuestas serológicas representativas se destacan como una fuente importante de datos para reducir la incertidumbre sobre políticas para las intervenciones farmacéuticas y no farmacéuticas una vez haya pasado la ola pandémica inicial.
- La vigilancia continua de la evolución temporal de la incidencia de casos graves H1N1pdm dará una imagen clara de la variabilidad subyacente en la transmisibilidad del virus durante cambios de comportamiento en la población, como las vacaciones escolares y otras intervenciones no farmacéuticas.
