# Supplementary material for: Studies Needed to Address Public Health Challenges of the 2009 H1N1 Influenza Pandemic: Insights from Modeling
Source: PLoS Med. 2010 Jun 1;7(6):e1000275. doi: 10.1371/journal.pmed.1000275 (PMC2879409; doi:10.1371/journal.pmed.1000275)
Supplement: Alternative Language Abstract S4 — Abstract translated into Finnish by TA. (0.03 MB DOC) [file pmed.1000275.s004.doc]

**Yhteenveto**

- Kun maailmanlaajuinen influenssa A(H1N1)v influenssapandemia etenee vuoteen 2010, merkittäviä menettelytapa haasteita tulee jatkossakin esille tulevien 12-18 kuukauden aikana.
- Osaamme odottaa kuutta kansanterveydellistä haastetta ja määrittää tiedot, joita tarvitaan julkisen terveydenhuollon päätöksentekoon: mittaus ikä-kohtaisesta tartunnan immuniteetistä, tarkasti määrittää tartunnan vakavuus, parantaa vakavien tapauksien hoitotuloksia, määrittää vastatoimien tehokkuuden, määrittää pandemian koko vaikutus kuolleisuuteen sekä nopeasti tunnistaa ja vastata antigeenien muutoksiin.
- Edustavat serologiset tutkimukset ovat tietolähteitä, joiden avulla voidaan vähentää epävarmuutta terveydenhuollon päätöksentekoon sekä lääkkeiden ja muiden vastatoimien kautta, kun ensimmäinen aalto on ohi.
- Jatkuva seuranta influenssa A(H1N1)v takapauksista antaa selkeän kuvan tartunnansuuruuden vaihtelusta väestön käyttäytymiseen koskevia muutoksia, kuten koulujen lomat ja muut lääkkeettömät vastatoimet.
