# Supplementary material for: Studies Needed to Address Public Health Challenges of the 2009 H1N1 Influenza Pandemic: Insights from Modeling
Source: PLoS Med. 2010 Jun 1;7(6):e1000275. doi: 10.1371/journal.pmed.1000275 (PMC2879409; doi:10.1371/journal.pmed.1000275)
Supplement: Alternative Language Abstract S5 — Abstract translated into French by J-CD. (0.03 MB DOC) [file pmed.1000275.s005.doc]

**Points clès**

- L'impact épidémiologique mondial de la grippe lièe à la souche pandémique 2009 va se prolonger en 2010 ce qui continuera à poser des défis de politique publique de santé dans les 12 à 18 mois prochains
- Dans cet article, nous anticipons six défis de santé publique et identifions les données nécessaires pour que la décision en santé publique : estimer l'immunité spécique à l'infection selon l'âge; quantifier de maière appropriée la gravité; améliorer les résultats de la prise en charge pour les formes sévères; estimer l'efficacité sur le terrain des interventions; préciser l'impact direct et indirect de la pandémie sur la mortalité, et identifier et répondre de manière réactive à l'émergence des variants antigéniques.
- Les enquêtes sérologiques sur des échantillons représentatifs de population sont des sources primordiales d'information pour éclairer les choix stratégiques en matière interventions pharmaceutiques et non pharmaceutiques après le passage de la première vague épidémique.
- Le maintien d'un suivi continu de l'incidence des formes sévères de grippe dues au virus H1N1pdm renseignera sur la variabilité sous-jacente de la transmissibilité du virus en lien avec les modifications de comportements à l'échelle des populations, tels que les vacances scolaires et d'autres interventions basées sur des mesures non pharmaceutiques.
