# Supplementary material for: Studies Needed to Address Public Health Challenges of the 2009 H1N1 Influenza Pandemic: Insights from Modeling
Source: PLoS Med. 2010 Jun 1;7(6):e1000275. doi: 10.1371/journal.pmed.1000275 (PMC2879409; doi:10.1371/journal.pmed.1000275)
Supplement: Alternative Language Abstract S6 — Abstract translated into Romanian by TA. (0.03 MB DOC) [file pmed.1000275.s006.doc]

**Puncte Sumar**

- Prin continuarea pandemiei gripale din 2009 si in provocările politice substanţiale vor continua pentru urmatoarele 12 la 18 luni
- Aici, putem anticipa şase provocări de sănătate publică şi identifica date care sunt necesare pentru a lua decizii de sănătate publică: măsurarea imunităţii la infecţii specifice vârstei- ; cuantificarea cu exactitate a severităţii; îmbunătăţirea rezultatelor de tratament pentru cazurile severe; cuantificarea eficienţei intervenţiilor; evidentierea în totalitate a impactului pandemiei asupra mortalităţii, precum şi identificarea şi răspunsul rapid la variantele antigenice.
- Studii serologice reprezentative sunt o sursă de date esentiale cu care se reduce incertitudinea din opţiunile politice pentru intervenţiile farmaceutice şi non-farmaceutice, după valul iniţial a trecut.
- Continuarea monitorizarii incidenţei cazurilor grave de H1N1pdm va oferi o imagine clară a variabilitatii in transmisibilitatea virusului în timpul schimbari de comportament la nivelul populaţiei, cum ar fi vacanţele şcolare şi alte intervenţii non-farmaceutice.
