# Supplementary material for: Studies Needed to Address Public Health Challenges of the 2009 H1N1 Influenza Pandemic: Insights from Modeling
Source: PLoS Med. 2010 Jun 1;7(6):e1000275. doi: 10.1371/journal.pmed.1000275 (PMC2879409; doi:10.1371/journal.pmed.1000275)
Supplement: Alternative Language Abstract S7 — Abstract translated into Swedish by TA. (0.03 MB DOC) [file pmed.1000275.s007.doc]

**Sammanfattning**

- Avsevärda utmaningar inom hälsobeslutsområdet kommer att komma de kommande 12 -18 månaderna, när det globala utbrottet av den nya influensan A(H1N1) pandemin fortsätter år 2010
- Vi räknar med sex utmaningar inom folkhälsoområden och identifierar de data som behövs för beslut inom folkhälsoområdet: mätning av åldersspecifik immunitet mot infektioner, exakt kvantifiera allvarlighet, förbättra resultaten angående behandlingen av svåra fall, kvantifiera effekten av interventioner, fånga hela effekten av pandemin på dödligheten, och snabbt identifiera och agera på antigenvariationer.
- Representativa serologiska undersökningar är en viktig källa för data som kan minska osäkerheten kring beslutsvalsalternativ för både pharmaceutiska och icke-pharmaceutiska interventioner, efter att den första vågen har passerat.
- Fortsätt övervakning av incidencen av allvarliga influensan A(H1N1) fall kommer att ge en tydlig bild av variationer i underliggande smittsamhet av viruset under befolkningen beteendeförändringar såsom skolsemestrar och andra icke-pharmaceutiska interventioner.
